# Application of Nanoparticles in the Treatment of Lung Cancer With Emphasis on Receptors

**DOI:** 10.3389/fphar.2021.781425

**Published:** 2022-01-10

**Authors:** Jingyue Wang, Tong Zhou, Ying Liu, Shuangmin Chen, Zhenxiang Yu

**Affiliations:** ^1^ Department of Cardiology, The First Hospital of Jilin University, Changchun, China; ^2^ Department of Endocrinology and Metabolism, The First Hospital of Jilin University, Changchun, China; ^3^ Department of Respiration, The First Hospital of Jilin University, Changchun, China

**Keywords:** lung cancer, nanoparticle, active targeting, receptors, biological ligands, drug delivery

## Abstract

Lung cancer is one of the malignant tumors that has seen the most rapid growth in terms of morbidity and mortality in recent years, posing the biggest threat to people’s health and lives. In recent years, the nano-drug loading system has made significant progress in the detection, diagnosis, and treatment of lung cancer. Nanomaterials are used to specifically target tumor tissue to minimize therapeutic adverse effects and increase bioavailability. It is achieved primarily through two mechanisms: passive targeting, which entails the use of enhanced penetration and retention (EPR) effect, and active targeting, which entails the loading recognition ligands for tumor marker molecules onto nanomaterials. However, it has been demonstrated that the EPR effect is effective in rodents but not in humans. Taking this into consideration, researchers paid significant attention to the active targeting nano-drug loading system. Additionally, it has been demonstrated to have a higher affinity and specificity for tumor cells. In this review, it describes the development of research into active targeted nano-drug delivery systems for lung cancer treatment from the receptors’ or targets’ perspective. We anticipate that this study will help biomedical researchers use nanoparticles (NPs) to treat lung cancer by providing more and novel drug delivery strategies or solid ligands.

## 1 Introduction

Cancer is a major cause of mortality worldwide, with over 200 distinct types, the most common of which is lung cancer, which is also the leading cause of cancer-related deaths. Lung cancer accounted for 11.6% of the 2.09 million new cancer cases diagnosed in 2018 and 18.4% of the 1.76 million cancer deaths. Among all malignant tumors, men have the highest incidence and mortality rates for lung cancer, while women have the third and second highest incidence and mortality rates for lung cancer, respectively (Bade and Dela Cruz, 2020). Lung cancer is classified as small cell lung cancer (SCLC) or non-small cell lung cancer (NSCLC), with about 80% of cases being NSCLC and 20% being SCLC. NSCLC can be divided into adenocarcinoma, squamous cell carcinoma, large cell carcinoma, and mixtures. SCLC is classified into three subtypes: small cell carcinoma, mixed small cell and large cell carcinoma, and mixed small cell carcinoma, each of which has a distinct therapeutic profile and clinical prognosis (Khanmohammadi et al., 2020). Lung cancer is often treated with surgery, chemotherapy, radiotherapy, and adjuvant therapy (Zappa and Mousa, 2016). Traditional chemotherapy treatments for lung cancer are unable to specifically target tumor cells and cause significant damages to normal cells, including bone marrow arrest, gastrointestinal reactions, and phlebophlogosis (Jin et al., 2018), thus limiting the development of anticancer drugs. In the 20th century, Paul Ehrlich, inspired by Karl Maria von Weber’s opera “DerFreischutz,” coined the term “magic bullet” for the first time and later expanded on the concepts of nanoparticles (NPs) and drug targeting in medicine (Kreuter, 2007). In recent years, advancements in drug nanotechnology have effectively overcome the drawbacks of traditional chemotherapy drugs. NPs have a small particle size, large specific surface area, and good biocompatibility and degradability. The critical point is that NPs may passively target tumor cells, and active targeting can be achieved by modifying the surface of NPs to enhance the therapeutic impact and decrease the toxicity of anticancer drugs (Ahmad et al., 2015).

**FIGURE 1:**
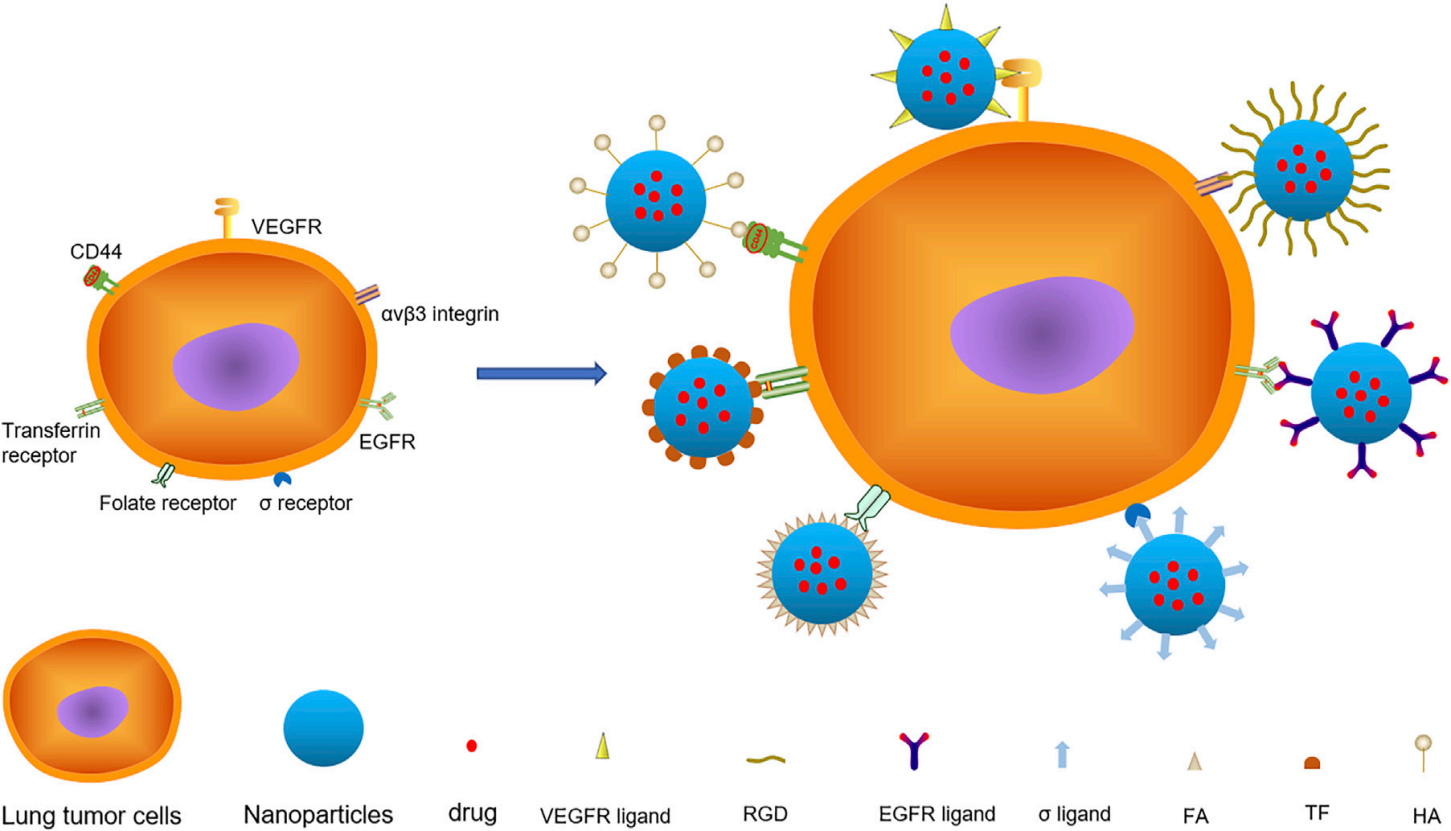
There are various targets on lung cancer cells, among which VEGFR, αvβ3 Integrin, EGFR, σ Receptor, Folate Receptor, Transferrin Receptor and CD44 are important targets. The nanoparticles with specific ligand structure loaded with anticancer drugs specifically bind to the receptors on the surface of lung cancer and deliver the drugs to the cells.

Nanomedicine is a relatively new form of treatment that focuses on replacing drug delivery and improving therapeutic effects while minimizing adverse effects on normal tissues (Markman et al., 2013). NPs used in the treatment of lung cancer can be divided into two categories: organic and inorganic NPs. The following are the major classes of organic NPs: 1) liposome-cholesterol and phospholipid-like biofilm-like NPs, 2) solid lipid NPs, 3)nanostructured lipid carriers-NPs mixed with solid lipids and liquid lipids, 4) polymeric NPs composed of polymers such as sodium alginate, chitosan, gelatin, polycaprolactone, polylactide, and polylactic acid, 5) Polymeric micelles-colloidal NPs composed of amphiphilic block copolymers, 6) dendrimer-highly branched, symmetrical, radiating NPs. Inorganic NPs are classified into three types: 1) magnetic NPs-superparamagnetic materials with size > 25 nm, 2) carbon nanotubes-hydrophobic tubular structure made by carbon atoms diameters between 4 nm and 100 mm, and 3) quantum dots-colloidal NPs with atomic properties (Sharma et al., 2019). There are three passive targeting techniques. One is to use tumors’ enhanced penetration and retention (EPR) effect to induce NPs to accumulate in tumor tissue, which does not work in humans (Danhier, 2016). The second technique is to use the acidic microenvironment of tumors to limit the action of NPs to acidic conditions (In and Nieva, 2015). Thirdly, tumor cells can be used to carry additional negative charges, since NPs are positively charged (Prabhu et al., 2015). Active targeting couples ligands to NPs via the receptors overexpressed explicitly on the surface of tumor cells or blood vessels, providing NPs with a more precise targeting effect than passive targeting. Additionally, the interaction of certain receptors and ligands has been shown to facilitate cell endocytosis and inhibit tumor multi-drug resistance (Bazak et al., 2015). Antibodies, fragments, aptamers (APTs), or small molecules are frequently used as ligands. Numerous previous studies (Landesman-Milo et al., 2015; Hussain, 2016; Mangal et al., 2017; Yu et al., 2017; Crintea et al., 2021) have described the characteristics and applications of current NPs and ligands for lung cancer treatment. Thus, this review mainly focused on several nanocarriers with active targeting functions for lung cancer treatment in detail from the receptors’ perspective. The studies related to the major biological receptors and their applications are summarized in Table 1. The combined application of biological ligands and NPs are summarized in Figure 1. The oncogenic signaling pathways and drugs targeting abnormal signals in lung cancer therapy are summarized in Table 2.

**TABLE 1:** Receptors: principal categories, functions, and applications.

Major categories of receptors	Functions and applications
VEGFR	Functions: increase vascular permeability; make lung cancer drug-resistant Tran et al. (2002), Herbst et al. (2005)
	• tLYP-1(NRP-1)enhanced the tumor inhibitory effect and reduced the side effects Jin et al. (2018)
	• Flk-1(VEGFR-2)enhanced the tumor inhibitory effect and reduced the side effects on heart and kidney Liu et al. (2011)
αvβ3 Integrin	Functions: promoting tumor angiogenesis and tumor metastasis Marelli et al. (2013)
	• GRGDSP(αvβ3 and α5β1)inhibited tumor growth and reduced side effectsBabu et al. (2017)
	• RGD (Integrin) improved the anti-tumor activity and delayed the release of loaded drugs Wang et al. (2018)
	• cRGD (Integrin) -PS-DOX was more likely to accumulate in tumors Zou et al. (2018)
EGFR	Functions: involve in tumor growth and progression, including proliferation, angiogenesis, invasion and metastasis Singh et al. (2016)
	• ER (EGFR) enhanced the targeting effect on PC-9 (Zhang et al., 2019)
	• EGF (EGFR) increased the distribution of NPs in tumor tissues, enhanced the uptake of NPs by lung cancer cells, and greatly enhanced the tumor-killing effect of drugs Zhang et al. (2016)
	• APT improved tumor inhibition, induced apoptosis, and had more minor side effects Lv et al. (2018)
σ Receptor	Functions: overexpressed in rapidly proliferating normal cells and cancer cells such as malignant melanoma, glioma, breast cancer, prostate cancer, SCLC and NSCLC van Waarde et al. (2015)
	• AA improved the delivery efficiency of siRNA by 9 times Yang et al. (2012)
	• AA can target the overexpressed σ receptor Xiong et al. (2016)
Folate Receptor	Functions: tumor tissue specificity; tumor marker
	• Improved the effectiveness and specificity of photodynamic therapy (PDT) Kato et al. (2017)
	• Den NPs:suitable carrier for co-delivery of siRNA and chemotherapeutic drugs in lung cancer cells Amreddy et al. (2018)
	• Efficacy of folate receptor-α (FRA)-targeted DOTAP Muralidharan et al. (2016)
	• FA modified amphiphilic PEG-PLGA copolymer NPs carried with CDDP and PTX He et al. (2015); also effective for the combined delivery of cisplatin and paclitaxel He et al. (2016)
Transferrin Receptor	Functions: expression level is higher in cells with a high proliferation rate, especially in tumor cells Daniels et al. (2006), Cheng et al. (2011), Dev and Babitt, (2017)
	• As a monitoring index of gambogic acid therapy sensitivity Zhu et al. (2009)
	• Combination of TFR and artemisinin could reduce small cell lung cancer drug resistance Sadava et al. (2002)
	• Thymoquinone-NP modified transferrin successfully couples two different miRNA pathways Upadhyay et al. (2019)
CD44	Functions: plays an important role in malignant tumor-related activities
	• HA-PCL- CAP nanoparticles identified the potential for the treatment of non-small cell lung cancer Parashar et al. (2019)

**TABLE 2:** Some drugs and their mechanism of action in lung cancer therapy.

Major categories of lung cancer drug therapy	Mechanism of action	Drugs
Chemotherapy	Platinum: By binding to the DNA in the nucleus, it destroys the DNA of tumor cells and induces apoptosis (Dilruba and Kalayda, 2016).	Platinum: cisplatin, carboplatin and oxaliplatin
	Taxane-type anticancer drugs: Through the unique microtubule stabilization mechanism, they act on the mitotic process, thus reducing the proliferation of cancer cells (Wen et al., 2016).	Taxane-type anticancer drugs: paclitaxel, docetaxel and cabazitaxel
	Gemcitabine: By infiltrating the intracellular DNA, it inhibits DNA synthesis, and ultimately leads to apoptosis (Mlak et al., 2016).	Gemcitabine
	Etoposide(VP-16): It acts on DNA topoisomerase II, causing DNA damage and promoting apoptosis (Nam et al., 2010).	Etoposide (VP-16)
Targeted therapy	Tyrosine kinase inhibitors (TKIs): It can inhibit the growth and induce apoptosis of tumor cells by inhibiting the two signal transduction pathways of RAS/RAF/MAPK/ERK and PI3K/AKT/mTOR. (Dienstmann et al., 2011).	Tyrosine kinase inhibitors (TKIs): gefitinib and erlotinib
	Receptor tyrosine kinase (RTK) inhibitor: Anti-tumor angiogenesis by selectively inhibiting vascular endothelial growth factor receptor-2 (EGFR-2) (Li et al., 2010; Ding et al., 2013).	Apatinib
Immunotherapy	Programmed cell death (PD)-1 immune checkpoint inhibitors: By combining with PD-1 receptor highly expressed on T cells, it can block the signal pathway induced by PD-L1 and PD-L2, and restore the function of T cells (Wang et al., 2014; Oya et al., 2017).	Nivolumab,pembrolizumab
Natural antitumor products	Combining the tumor targeting carriers with natural anti-tumor drugs in an appropriate way can improve the anti-tumor efficacy (Jin et al., 2018).	Parthenolide,ginsenoside compound K
	• Parthenolide: It can achieve anti-tumor effect by inhibiting B-Raf/MAPK/Erk signaling pathway, inhibiting NF-κB activation, and inhibiting PI3K/AKT signaling pathway (Jeyamohan et al., 2016; Kim et al., 2017; Lin et al., 2017).	
	• CK: It can induce apoptosis through glycogen synthase kinase 3β(GSK3β) signaling pathway and regulating reactive oxygen species (ROS). It can inhibit angiogenesis by inhibiting sphingosine kinase -1 (Kim et al., 2013; Shin et al., 2014; Kwak et al., 2015)	

## 2 Biological Receptors and Their Applications for Nanoparticles

### 2.1 Vascular Endothelial-Derived Growth Factor Receptors

Due to gene mutation and tissue hypoxia in lung cancer, the expression level of vascular endothelial-derived growth factor (VEGF) is increased by hypoxia-inducible factor-1α (HIF-1α) and matrix metalloproteinase (MMP), resulting in an overexpression of vascular endothelial-derived growth factor receptors (VEGFRs) in lung cancer cells and endothelial cells (Prabhu et al., 2015). The primary function of VEGFs is to induce angiogenesis, chemotactic endothelial cells and increase vascular permeability by binding and activating VEGFRs signal cascade. Additionally, VEGF can promote lung cancer metastasis and induce survivin expression, conferring lung cancer drug-resistant (Tran et al., 2002; Herbst et al., 2005). VEGFs are a family of glycoproteins that include VEGF-A, VEGF-B, VEGF-C, VEGF-D, VEGF-E, and placenta growth factor (PGF). VEGFRs have several receptor subtypes: VEGFR-1/Flt-1, VEGFR-2/Flk-1/KDR, VEGFR-3/Flt-4, neuropilin-1(NRP-1), and neuropilin-2(NRP-2), among which VEGFR-2 is the most important and prevalent subtype. VEGFRs consist of an extracellular ligand-binding domain, a transmembrane domain, and a cytoplasmic domain encoding a tyrosine kinase. NRPs consist of extracellular domains, single transmembrane domains, and short intracellular domains lacking intrinsic catalytic function (Frezzetti et al., 2017). VEGFRs are overexpressed on the surface of a wide range of tumor cells and *in situ* tumor neovasculature, making them prospective targets for “double targeting” (tumor and vascular targeting) tumor therapy (Liu et al., 2011). VEGFRs, in particular, is the better target for overexpression on the cell membrane surface (Liu et al., 2011).

Jin et al. (2018) designed a new type of liposome nanoparticles with an active targeting function for loading parthenolide and ginsenoside compound K (CK) for the treatment of NSCLC. The NPs used tumor homing peptide tLYP-1 (sequence CGNKRTR) as the ligand and had a hydrophilic PEG shell. The particle size was 188 nm. The encapsulation efficiency of CK and parthenolide was 83.4 and 70.7%, respectively, while the drug loading efficiency was 14.8 and 2.9%, respectively. The PEG shell of new NPs can improve its stability, prolong cycle time, and increase solubility. tLYP-1 can enhance its tissue penetration ability, selectively target the over-expressed NRP-1 on the surface of lung cancer cells, and enter the cells via receptor-mediated endocytosis, causing mitochondrial swelling and apoptosis, increasing the level of reactive oxygen species and inducing apoptosis. *In vivo* studies illustrated that the presence of tLYP-1 can undoubtedly increase the tumor inhibitory effect while minimizing adverse effects by increasing the selectivity of the tumor. It also suggests that researchers can further develop this new strategy for the effective treatment of cancer by using the combination of low-toxic natural products. Liu et al. (2011) developed a nanostructured lipid carrier with active targeting properties for docetaxel (DTX) loading. They studied its uptake in tumor and endothelial cells, as well as its therapeutic effect *in vivo* and biological distribution. Nanostructured lipid carriers (NLC) was found to be linked to the ligand (VEGFR-2 antibody) through DSPE-PEG-NH2. The average particle size of targeted NLC (tNLC) was 68.70 ± 2.07 nm; the encapsulation efficiency of DTX was 98.43 ± 0.51%; the drug loading rate was 5.55 ± 0.06%, and the average ligand coupling efficiency was 3.34 ± 2.63%. The findings indicated that tNLC first accumulated in tumor tissues via the EPR effect. It was then internalized by tumor cells and endothelial cells via the specific binding of Flk-1 with VEGFR-2, resulting in effective anti-tumor activity. tNLC has little influence on the growth of A549 and HUVEC cells, however, its polyethylene glycol coating can prevent interaction with serum proteins. Flk-1 can undoubtedly boost the tumor inhibitory effect while minimizing adverse effects by binding specifically on and internalization of tumor cells and tumor microvasculature. This study advances the targeted therapy strategy using VEGFR-2, but the precise metastasis mechanism of tNLC remains to be further studied.

Tumor neovascularization not only supplies nutrients to the tumor but also acts as a conduit for tumor diffusion and metastasis. VEGFR inhibition as a target for angiogenesis has become a research hotspot in tumor therapy. The purpose of this review was to summarize previous research on active targeted therapy for lung cancer using VEGFR ligands and NPs. A comparison is made between the new active targeting strategies for targeted-NPs (tNPs) (Table 3). More NPs-targeted drugs will be introduced to anti-lung cancer clinical therapy in the future, providing patients with more precise treatment options with fewer adverse effects.

**TABLE 3:** VEGFR antibody for active targeting of nanoparticle drug delivery systems.

Types	Encapsulation percentage,EN%	Loading efficiency,LE%	Size (nm)
tLYP-1-PEG-NP Jin et al. (2018)	CK:83.4	14.8	188
	Parthenolide:70.7	2.9	
Flk-1-DSPE-PEG-NH2-NLC Liu et al. (2011)	CTX:98.43 + 0.51	3.34 ± 2.63	168.70 ± 2.07

### 2.2 αvβ3 Integrin

Integrin is a transmembrane glycoprotein heterodimer composed of 18 α and 8 β subunits. There have been 24 distinct subtypes identified thus far, 11 of which specifically bind to the arginine-glycine-aspartic acid (Arg-Gly-Asp, RGD) sequence. The RGD sequence widely exists in extracellular matrix proteins, such as collagen, fibronectin, fibrinogen, laminin, von Willebrand factor, thrombospondin, osteopontin, and vitreous connective protein. Combining the extracellular domain of integrin with an extracellular matrix protein alters the conformation of its transmembrane domain and intracellular domain connected to the actin skeleton, hence regulating cell adhesion, migration, differentiation, proliferation, and survival (Aksorn and Chanvorachote, 2019). avβ3 integrin subtype is highly overexpressed on the surface of lung cancer cells and endothelial cells, promoting tumor angiogenesis and metastasis. Because the RGD sequence can specifically bind to αvβ3 integrin, numerous RGD peptides have been developed. avβ3 integrin biological and kinetic features can be altered by cyclizing, connecting other amino acids on both sides, and altering the stereo configuration or N-methylation. RGD peptides can improve the adherence of nanomaterials to lung cancer and mediate endocytosis into target cells (Marelli et al., 2013).

Babu et al. (2017) developed an RGD peptide modified polylactic acid-glycolic acid (PLGA)- chitosan-based nanoparticle system (CSNP)-RGD, loaded with paclitaxel (PTX) and used for NSCLC targeted drug delivery. The NPs were mainly drug-loaded PLGA, coated with positively charged chitosan, and linked to linear RGD peptides (GRGDSP). Chitosan can improve the stability of particles, control drug release, and increase adhesion. GRGDSP is a linear peptide with strong adhesion that recognizes the cell surface integrins αvβ3 and α5β1. PLGA-CSNP-RGD has an average particle size of 217 nm; the average entrapment efficiency of PTX is 93.7%, and the drug loading rate is 6.5%. The findings indicate that NPs have favorable physical and chemical properties and that NPs without drugs exhibit no apparent cytotoxicity. PTX-PLGA-CSNP-RGD is extremely selective for NSCLC cells such as A549 and H1299, which are overexpressed by integrin αvβ3. It penetrates cells via endocytosis mediated by integrin αvβ3, which inhibits the G2/M cell cycle and induces apoptosis of tumor cells, but has almost no toxicity to normal bronchial epithelial cells. GRGDSP has been shown to suppress tumor growth and alleviate adverse effects *in vivo*. This study shows PLGA-CSNP-RGD is expected to become a tool for priority drug delivery in lung cancer cells. And what deserves the attention of the researchers is that different normal cell lines need to be wisely evaluated when studying the efficacy of targeted nano-drug delivery systems in the treatment of cancer. Wang et al. (2018) synthesized RGD-modified lipid polymer NPs loaded with PTX and cisplatin (CDDP) (RGD-ss-PTX-CDDP LPNs) and investigated their anti-lung cancer effect in lung cancer cells and tumor-bearing animal models. RGD-ss-PTX-CDDP LPNs are mostly composed of PLGA loaded with PTX and CDDP and coated with soybean lecithin (SL). PTX is linked to the RGD peptide via PEG disulfide bonds, and disulfide bonds utilize a high concentration of glutathione (GSH) in tumor cells to allow specific intracellular drug release. The particle size was 191.3 ± 5.3 nm; the zeta potential was -37.2 ± 3.9 mV; the drug loading rates of PTX and CDDP were 5.4 ± 0.6% and 12.3 ± 1.1% respectively; the encapsulation rates were 85.3 ± 3.3% and 82.7 ± 4.1%, respectively. The results indicated that the NPs’ properties were stable and the structure remained unchanged after 30 days in phosphate buffered saline (PBS) and 5 days in the culture medium. At 100 μg/ml, the drug-free NPs exhibited no apparent cytotoxicity and rarely accumulated in normal tissues. RGD can enhance anti-tumor activity and delay drug release. This study provides a scheme for the preparation of specific RGD modified, redox sensitive, prodrug-based lipid polymer NPs, which can be used in the synergistic combination chemotherapy of PTX and CDDP. RGD-ss-PTX-CDDP LPNs has synergistic anti-tumor effect and low systemic toxicity. The resulting system may become a promising targeted nano-drug for the treatment of lung cancer. Zou et al. (2018) synthesized the cyclic RGD peptide disulfide cross-linked polymer doxorubicin (cRGD-PS-DOX) and studied its targeted therapeutic effect on NSCLC. cRGD-PS-DOX utilizes PEG-P (TMC-DTC), loaded with DOX, as the primary linking ligand cRGD. The particle size of cRGD-PS-DOX was 96nm and the drug loading rate of DOX was 15.2%. It was determined that the release of cRGD-PS-DOX was less than 15% at 37°C, pH = 7.4, and GSH concentration = 2 m within 24 h. The release amount increased with the increase in GSH concentration, demonstrating that NPs had excellent stability in circulation and the capacity to deliver drugs rapidly into cells. The 3-(4,5-dimethylthiazol-2-yl)-2,5-diphenyltetrazolium bromide (MTT) assay demonstrated that drug-free NPs were non-toxic to A549 cells, but drug-loaded NPs had a strong inhibitory effect. Flow cytometry analysis revealed that cRGD-PS-DOX had stronger DOX·HCl intensity than PS-DOX when A549 cells with αvβ3 integrin overexpression were treated respectively. In MCF-7 cells with decreased αvβ3 integrin expression, the two intensities were comparable, demonstrating that cRGD-PS-DOX exhibited a high degree of specificity for NSCLC. Pharmacokinetics analysis revealed that cRGD-PS-DOX accumulates more readily tumors than in normal tissues.

To date, several RGD peptides and their analogues have been developed for targeting integrin αvβ3. However, there are certain drawbacks, including low affinity, poor specificity, and so on. Therefore, the combination of NPs with targeted peptides is critical for lung cancer treatment. A comparison of the tNPs listed above was performed (Table 4).

**TABLE 4:** avβ3 antibody for active targeting of nanoparticle drug delivery systems.

Types	Encapsulation percentage,EN%	Loading efficiency,LE%	Size (nm)
PLGA-CSNP-RGD Babu et al. (2017)	PTX 93.7	6.5	217
RGD-ss-PTX/CDDP LPNs Wang et al. (2018)	PTX 85.3 ± 3.3	5.4 ± 0.6	191.3 ± 5.3
	CDDP 82.7 ± 4.1	12.3 ± 1.1	
cRGD-PS-Dox Zou et al. (2018)		15.2	96

### 2.3 Epidermal Growth Factor Receptor

The epidermal growth factor receptor (EGFR) is a member of the class I transmembrane receptor tyrosine kinase superfamily, which is composed of EGFR/ERBB1/HER1, ERBB2/HER2, ERBB3/HER3, and ERBB4/HER4. EGFR is composed of an extracellular ligand-binding region, a transmembrane region, and an intracellular kinase region, which plays an essential role in the physiological process of cell growth, proliferation, and differentiation. EGFR is widely distributed on the surface of mammalian epithelial cells, fibroblasts, glial cells, keratinocytes, etc., and is overexpressed in many cancers. Although the EGFR gene is not overexpressed in SCLC, it is overexpressed in more than 60% of NSCLC (Zhang et al., 2010). The EGFR may bind to seven different ligands: EGF, transforming growth factor-alpha (TGFA), heparin-bound EGF-like growth factor (HBEGF), betamycin (BTC), amphotericin (AREG), insulin (EREG), and epigenetic gene (EPGN). EGF, TGFA, HBEGF, and BTC are considered high-affinity ligands, while AREG, EREG, and EPGN are low-affinity ligands. These ligands activate EGFR to stimulate intracellular signaling processes involved in tumor growth and progression, including proliferation, angiogenesis, invasion, and metastasis (Singh et al., 2016). In addition, EGFR tyrosine kinase inhibitors (EGFRTKIs) such as erlotinib (ER), gefitinib, and afatinib are often utilized targeting ligands (Zhang et al., 2019). Additionally, the anti-EGFR APT is a frequently used targeting ligand (Lv et al., 2018).

Kim and Huang (2012) studied a peptide-based therapy aimed at blocking intracellular protein-protein interactions in EGFR signal transduction. They also evaluated a targeted lipid carrier system capable of delivering peptides to intracellular targets in human cancer cells, thereby validating the concept of intracellular peptide-mediated cancer therapy delivered via well-designed NPs. Zhang et al. (2019) successfully synthesized a redox/pH double reaction nano thermosensitive agent (ECMI) for targeted molecular imaging and synergistic EGFR mutant lung cancer treatment. ECMI utilizes mesoporous silica (MSN) as the main body, supports indocyanine green (ICG) through electrostatic interactions, and fills the pores of MSNs with ZnO quantum dots (QDs). To form a shell, the drug-loaded MSNs were coated with chitosan (Cs) modified by ER, a cross-linked disulfide ligand. The diameter of ECMI was 220.0 ± 3.5 nm; polydispersity indexes (PDI) was 0.41 ± 0.03; Zeta potential was −18.5 ± 1.7 mV; drug loading rate was 15 ± 2 μg/mg, and ER conjugation efficiency was 35 ± 1 μg/mg. *In vitro* and *in vivo* studies indicated that EMCI has good biocompatibility and degradability and can accumulate in wild-type A549, ER-sensitive PC-9, and ER-resistant H1975 tumor cells. In PC-9 cells, the average fluorescence intensity of ECMI is more potent than that of CMI, indicating that ER can significantly enhance the targeting effect on PC-9. Zhang et al. (2016) successfully developed a PEG- PLGA-PLL targeting nanoparticle (EGF-PEAL NPs) using EGF as a ligand and loaded DOX and Bcl-2 small interfering RNA (siRNA), respectively, to study the synergistic effect anti-lung cancer effect of DOX-EGF-PEAL NPs and BCL-2-EGF- PEAL NPs. Particle sizes of DOX-EGF-PEAL NPs and Bcl-2-EGF-PEAL NPs were 203.7 ± 7.42 nm and 206.5 ± 6.37 nm; Zeta potentials were 3.5 ± 0.88 mV, and 2.1 ± 1.24 mV; PDI were 0.182 and 0.217, respectively. The results indicated that EGF-PEAL NPs were biodegradable and compatible with H1299 cells and exhibited low toxicity. EGF-PEAL NPs loaded with DOX or Bcl-2-siRNA showed a drug slow-release mode. EGF can significantly boost the dispersion of NPs in tumor tissues, enhance the uptake of NPs by lung cancer cells, and greatly enhance the tumor-killing effect of drugs. At the same time, EGF can decrease the concentration of NPs in the liver, spleen, and kidney and reduce the phagocytosis of NPs by the reticuloendothelial system and mononuclear phagocytic system. AnnexinV/PI staining showed that the combination of DOX-EGF-PEAL NPs and Bcl-2-EGF-PEAL NPs may have the most significant therapeutic effect. Lv et al. (2018) synthesized targeted NPs (AP/ES) with anti-EGFR,APT as ligand, polyamine dendrimer (PAMAM) as the main body, and simultaneously loaded ER and Survivin-short hairpin (shRNA), and studied the therapeutic effects of AP/ES and AP/ES combined with chloroquine (CQ) on EGFR mutant NSCLC. AP/ES exhibits favorable physical and chemical properties, including average particle size of 383.1 ± 0.4 nm, zeta potential of 12.1 ± 0.1 mV, ER drug loading rate of 10.4 ± 0.7%, encapsulation efficiency of 18.7 ± 0.4%, and PDI of 0.27 ± 0.01. The results demonstrate that anti-EGFR APT modified NPs can specifically recognize, bind, and deliver drugs and genes to EGFR overexpressed in NSCLC cells simultaneously. They can significantly improve tumor inhibition, induce apoptosis, and have fewer adverse side effects when compared with non-targeted NPs. The combined use of CQ and AP/ES improves tumor microcirculation and gene transfection efficiency, hence promoting drug delivery and efficacy of AP/ES, and has shown promising results in patients with ER-resistant NSCLC.

With the continuous development of tumor cytology and molecular biology, the potential of EGFR as a targeted therapy for lung cancer has been shown. However, several issues and technological difficulties persist. A comparison of these tNPs was performed (Table 5).

**TABLE 5:** EGFR antibody for active targeting of nanoparticle drug delivery systems.

Types	PDI	Zeta potential (mV)	Size (nm)
ECMI Zhang et al. (2019)	0.41 ± 0.03	−18.5 ± 1.7	220.0 ± 3.5
Dox-EGF-PEAL NPs	0.182	3.5 ± 0.88	203.7 ± 7.42
Bcl-2-EGF-PEAL NPs Zhang et al. (2016)	0.217	2.1 ± 1.24	206.5 ± 6.37
AP/ES Lv et al. (2018)	0.27 ± 0.01	12.1 ± 0.1	383.1 ± 0.4

### 2.4 σ Receptor

σ receptor is one of the important targets of lung cancer, which is classified into two subtypes: σ1 and σ2. They are not genetically related, but their functions are similar. Recent studies have shown that the σ1 receptor is a single-channel transmembrane protein, whereas the σ2 receptor is TMEM97, a four-channel transmembrane protein. σ receptor is mainly expressed in the intracellular membrane system, with a small amount expressed in the cell membrane (Schmidt and Kruse, 2019). Additionally, the σ receptor, especially σ2 receptor, is rapidly overexpressed in proliferating normal cells and cancer cells such as malignant melanoma, glioma, breast cancer, prostate cancer, SCLC, and NSCLC. Surprisingly, σ ligands can only cause cancer cell death (van Waarde et al., 2015). σ1 ligand can reduce the invasiveness of tumors, while σ2 ligand can mediate cancer cells’ death via autophagy, cell cycle interference, and apoptosis (Ramzy et al., 2017). σ1 ligand can be classified into Gilligan and Glennon/Ablordeppey models, while σ2 ligand can be classified as amine derivatives with limited conformation, indole analogs related to Siramesine, and amine derivatives with flexible conformation (Georgiadis et al., 2017). Anisamide (AA) is a benzamide derivative with a small molecular weight and is the most common σ ligand.

Yang et al. (2012) developed lipid-calcium-phosphate (LCP) targeting NPs with AA as the ligand and simultaneously loaded three siRNAs (HDM2: c-myc: VEGF = 1: 1: 1, weight ratio) to study the therapeutic effect of AA-siRNA-LCP NPs on NSCLC. With calcium phosphate (CaP)-siRNA as the core, the NPs were stabilized with dioleoylphosphatidic acid (DOPA), coated with 1,2-dioleoyl-3-trimethylammonium-propane chloride salt (DOTAP), and further linked to 2-Distearoryl-sn-glycero-3-phosphoethanol-amine-N-[methoxy (polyethyleneglycol-2000) ammonium salt (DSPE-PEG_2000_)-AA, and reported a particle size of 38.6 ± 3.6 nm and Zeta potential of 29.1 ± 1.3 mV. The results showed that AA-LCP NPs have a high affinity for nucleic acid. They are biocompatible and biodegradable, and dissolve rapidly in acidic pH. AA-LCP NPs can significantly reduce the reticuloendothelial system (RES) uptake, increase tumor accumulation, and effectively deliver siRNA to NSCLC cells overexpressing the σ receptor. AA can improve the delivery efficiency of siRNA by 9 times, which significantly enhance the inhibition of HDM2/c-myc/VEGF expression, inhibit tumor proliferation and angiogenesis, and induce tumor apoptosis. Targeted LCP is a promising carrier that can deliver multiple siRNA to NSCLC, realize multi-target blocking, and effectively inhibit tumor growth, which suggests pooled siRNA formulated in targeted LCP may become a useful tool for NSCLC therapy. Xiong et al. (2016) designed a cationic liposome (LP) targeted nanocomposite with AA as the ligand and used it to load cisplatin (CDDP) and metformin. Using H460 cell xenotransplantation as a model, the synergistic anticancer effect on NSCLC was studied. Electrostatic complexation of anionic polyglutamic acid (PGA) and cationic polymer metformin (Polymet) created the core of NPs, which is covered with polyglycolic cationic LP and coupled to the ligand AA through DSPE-PEG. The NPs had a particle size of 145 ± 1 nm and a zeta potential of 49 ± 1 mV. The results showed that NPs had a high degree of stability, biocompatibility, and biodegradability. While the combination of CDDP and PGA inhibits medication release and impairs the therapeutic effect, NPs greatly inhibit RES absorption and promote tumor accumulation. AA can target the overexpressed σ receptor, and Polymet and CDDP work synergistically. These improvements not only render drug-loaded NPs statistically equivalent to or even better than free CDDP in terms of effectiveness but also significantly lower systemic toxicity and CDDP resistance. Haloperidol (Hal) is a ligand targeting σ2 receptor overexpressed in NSCLC. Varshosaz et al. (2015) synthesized Hal targeting bovine serum albumin (BSA) NPs for pulmonary delivery of DOX. The tNP size was 218 nm, the zeta potential was −25.4 mV, the drug entrapment efficiency was 89%, and the 2 h release rate was 56%.

There have been few studies on the σ receptor ligands to far, and the most often used ligand is AA. Table 6 compares the binding of AA to NPs. In the future, more researches on the mechanism which causes lung cancer are required to identify additional ligands.

**TABLE 6:** σ Receptor antibody for active targeting of nanoparticle drug delivery systems.

Types	Size (nm)	Zeta potential (mV)
AA-siRNA-LCP NPs Yang et al. (2012)	38.6 ± 3.6	29.1 ± 1.3
AA-Cationic-LP NPs Xiong et al. (2016)	145 ± 1	49 ± 1
Hal-BSA NPs Varshosaz et al. (2015)	218	−25.4

### 2.5 Folate Receptor

Folate acid (FA) is a water-soluble vitamin commonly known as vitamin B9. It is required for the body to utilize carbohydrates and amino acids in cell division and growth, nucleic acid, and protein synthesis. Together with vitamin B12, it is known that FA functions *in vivo* as tetrahydrofolate acid and contributes to the synthesis and transformation of purine and pyrimidine nucleotides, aids in protein metabolism, and promotes the formation and maturation of erythrocytes. Due to the low relative molecular weight of FA, it is easy to modify and penetrate tumor cells and has low immunogenicity. FA offers several benefits, including a short time to reach the target, fast blood clearance, intense penetration, low human immune response, and so on (Prabhu et al., 2015; Sharma et al., 2019). FA be utilized as a tumor-targeting agent and is frequently used in the clinic. The folate receptor (FR) is a 38 kDa glycosyl-phosphatidylinositol binding glycoprotein, which often binds to FA, FA coupling drugs or FA anchored nanocarriers with high affinity, and internalizes FA into cells by mediating the endocytosis of FR. Studies have confirmed that FR is highly expressed on the surface of some tumor cells but has no or little expression in normal tissues, indicating that it has a reasonable tumor tissue specificity and is the most extensively researched tumor marker. In recent years, FRs have gained increasing interest in the domains of targeted drug delivery, cancer, and immunotherapy of rheumatoid arthritis. It is well established that FRs exist in three isomers: FRα, FR β, and FR γ (Bazak et al., 2015). FR α is highly expressed in epithelial cell line tumors, such as ovarian cancer, colorectal cancer, lung adenocarcinoma, etc. FR β is often highly expressed on the surface of tumor cells derived from non-epithelial tissues, such as sarcoma and acute myeloid leukemia. It is a potential target for FR-mediated targeted myeloid leukemia therapy, and FR γ can be used as a serum marker for lymphoma because it is not detected in normal serum (Jin et al., 2018). With the increasing understanding of FRs on the cell membrane, an FA-binding protein that is anchored to the cell membrane by phosphatidylinositol and can be removed by specific phospholipase C or D, it was discovered that the activity and number of FRs on the membrane surface of many types of tumor cells (such as ovarian cancer, colorectal cancer, breast cancer, lung cancer, and renal cell carcinoma) were significantly higher than those of normal cells. It lays a foundation for the study of drug targeting of tumor cells mediated by FA. Due to the high level of expression of FR on the surface of a variety of cancer cells, particularly the surface of malignant epithelial tumors, it is critical in the targeted therapy of nasopharyngeal carcinoma, lung cancer, colon cancer, gastric cancer, and gynecological tumors. By precisely binding to the overexpressed FR on the surface of tumor cells, the FR-mediated targeted drug delivery system can increase the drug’s toxicity to the target and reduce the side effects. However, at present, the targeting evaluation of this kind of targeted drug delivery system is mainly based on *in vitro* cytotoxicity test and cell uptake rate test; there is a dearth of experimental data in animals, and the targeted therapeutic effect in humans people requires additional investigation.

Kato et al. (2017) found that novel porphyrin lipid NPs targeting folate receptor 1 (FOLR1) can improve the effectiveness and specificity of photodynamic therapy (PDT), which is helpful for the minimally invasive intervention of peripheral lung cancer and advanced lung cancer metastatic lymph nodes. They constructed subcutaneous and *in situ* lung cancer models in mice and constructed targeted NPs with FA and porphysomes. Porphysomes are unique NPs made of two naturally occurring molecules (chlorophyll and lipids) that have the potential to be used in a wide range of biological photon applications. This nanostructure is like a miniature colorful water balloon through which the drug-loaded NPs may be delivered to the tumor for targeted treatment. These NPs exhibit a broad range of variable absorptivity, structure-dependent fluorescence self-quenching, and unique photothermal and photoacoustic properties (Lovell et al., 2011). Amreddy et al. (2018) demonstrated that the dendrimer (Den) nanoparticle system targeting the FR-α is a suitable carrier for co-delivery of siRNA and chemotherapeutic drugs in lung cancer cells. Den-Polyethlyeneimine (PEI)- cis-diamine platinum (CDDP)-siRNA-FA NPs have low molecular weight (800 MW), well-dispersed spherical particles less than 10 nm in size, encapsulation efficiencies for CDDP 40.52 ± 4.18%, zeta potential +17.2 mV. Muralidharan et al. (2016) established the effectiveness of FR-α (FRA)-targeted DOTAP: Human lung cancer cells were treated with cholesterol lipid NPs containing HuR siRNA (HuR-FNP). HuR-FNP particle size had a diameter of 303.3 nm. Compared with normal lung fibroblast (CCD 16) cells, human lung cancer (H1299) cells had significantly higher uptake of FNP, indicating the existence of receptor dose effect. HuR-FNP zeta potential was 4.3 mV. About 39% of siRNA was released in an acetic acid buffer for the first hour. He et al. (2015) found that the FA modified amphiphilic PEG-PLGA copolymer NPs combined with CDDP and PTX may be used to guide efficient and safe cancer chemotherapy, particularly in tumors with high FA receptor expression. The particle size of Co-FA-NPs (CDDP: PTX = 2:1) was 171.36 ± 8.67 nm, PDI was 0.133 ± 0.008, and zeta potential was 21.50 ± 0.88 mV. The targeted NPs are also effective for the combined delivery of cisplatin and paclitaxel in the treatment of non-small cell lung cancer. Additionally, the tNPs are also effective for delivering cisplatin and paclitaxel in the treatment of NSCLC (He et al., 2016).

Table 7 compares FA binding to NPs. FA combined with NPs provides a new solution to toxicity and side effects, treatment drug resistance, and poor detection strategies of cancer treatment, making it a promising theragnostic ligand.

**TABLE 7:** Folate Receptor antibody for active targeting of nanoparticle drug delivery systems.

Types	Size (nm)	Zeta potential (mV)
Den-PEI-CDDP-siRNA-FA NPs Amreddy et al. (2018)	<10	17.2
HuR-FNP Muralidharan et al. (2016)	Diameter 303.3	4.3
Co-FA-NPs He et al. (2015)	171.36 ± 8.67	21.50 ± 0.88

### 2.6 Transferrin Receptor

Human transferrin (TF) is an iron-binding protein with a molecular weight of 79.57 kDa. It may bind to the transferrin receptor (TFR) to transfer iron absorbed via the digestive tract and iron released by red blood cell degradation into bone marrow in the form of TFR-Fe3+ complex to generate mature red blood cells. Additionally, it is capable of transporting iron into cells that express TFRs. TF is biodegradable, non-toxic, non-immunogenic, and may be used to target particular sites via TFR expression on the cell surface (Vaidya and Vyas, 2012). TFR is expressed at a modest level in all normal nucleated cells. Studies have shown that the expression level is higher in cells with a high proliferation rate, especially in tumor cells, such as liver cancer, chronic lymphocytic leukemia, lung cancer (Daniels et al., 2006; Cheng et al., 2011; Dev and Babitt, 2017), as evidenced by increased cell membrane expression and blood concentration (Feelders et al., 1999). This phenomenon is referred to as iron addiction (Manz et al., 2016), as iron is required for a variety of key biological functions, including cell proliferation and growth (Dev and Babitt, 2017). Because TFR is overexpressed in a variety of tumor cells, TF/TFR-mediated cell events have been used to deliver therapeutic drugs to malignant tumor cells (Tros de Ilarduya and Düzgüneş, 2013).

Zhu et al. (2009) found that the histopathological quantification of TFR expression in lung cancer may be employed in clinical practice to monitor gambogic acid treatment susceptibility. Multi-drug resistance is a fundamental problem in SCLC. Sadava et al. (2002) found that the combination of TFR and artemisinin could reduce small cell lung cancer drug resistance. The low molar concentration of artemisinin after pretreatment could kill the cells in SCLC. Upadhyay et al. (2019) found that thymoquinone-NP modified transferrin successfully couples two distinct miRNA pathways, enhances the apoptosis and death cascade of extremely lethal NSCLC cells, and limits these migration cells without producing any significant toxicity like the widely used combination of chemotherapeutic drugs.

In a nutshell, the transferrin receptor plays an important role in the abnormal iron metabolism of lung cancer cells. Although its mechanism is not fully understood, we can reduce the iron intake of cancer cells through transferrin receptors, thus interfering with the iron metabolism of lung cancer cells and limiting their proliferation, thereby enabling novel therapy options for the treatment of lung cancer diseases. Transferrin receptors will have a good application prospect in the treatment of tumor diseases.

### 2.7 CD44

CD44 is a membrane-bound glycoprotein that plays an important role in malignant tumor-related activities. It has been demonstrated that it can be used as a cancer stem cell (CSC)/tumor-initiating cells (TIC) marker (Fasano et al., 1997; Du et al., 2008; Ghosh et al., 2012). CSC theory suggests that cancer is composed of tumor cell subsets with the characteristics of stem cells or progenitor cells. These cells are capable of initiating tumor formation and differentiation via a variety of effective pathways and exhibiting a high level of resistance to conventional chemotherapy (Leung et al., 2010). The receptor is overexpressed in various solid tumors, such as pancreatic cancer, breast cancer, and lung cancer (Mattheolabakis et al., 2015). In mechanism, the invasive and metastatic growth of cancer cells can be mediated by the interaction between CD44 on the cell surface and extracellular matrix components such as hyaluronic acid and then induce changes in the cytoskeleton of cancer cells (Marhaba et al., 2008). CD44 is also involved in many signal cascades that mediate tumor enhancement. As a co-receptor with adjacent EGFR or other ErbB family receptor tyrosine kinases, it can indirectly activate cell proliferation pathways through ligands (Bourguignon et al., 2007). Additionally, it can activate anti-apoptotic pathways, such as phosphatidylinositol-3-kinase (PI3K)/protein kinase B (AKT) signal cascade (Bourguignon et al., 2007) and B lymphocyte tumor-2 gene (Bcl-2) and Bcl-xl transcription to enhance the survival ability of tumor cells (Madjd et al., 2009). According to research on SCLC, activation of CD44-MAPK-PI3K signal transduction results in increased expression of urokinase plasminogen activator (uPA)/uPA receptor (uPAR) and multi-drug resistance gene (MDR) 1, resulting in increased invasiveness and multi-drug resistance phenotype of lung cancer cells (Gutova et al., 2007).

Jeannot et al. (2016) proved that the size of hyaluronic acid (HA) NPs plays an important role in cellular uptake and biological distribution. Small NPs exhibit a positive targeting effect on tumors overexpressing CD44, implying that they might be employed as drug delivery systems. Poly-ε-caprolactone (PCL) is a commonly used polymer. Due to its biocompatibility and biodegradability, it has become the preferred targeted drug delivery system for scientists (Amreddy et al., 2017). The anticancer activity of Capsaicin (CAP) has been extensively examined for its anticancer activity in a variety of cancers, including lung cancer, where it has been shown to operate as an anticancer and anti-proliferative agent on a variety of human cancer cell lines (Galano and Martínez, 2012). Parashar et al. (2019) demonstrated that HA-PCL- CAP NPs identified the potential for the treatment of NSCLC and showed enhanced cytotoxicity, anti-proliferation, and apoptosis properties of CAP in A549.

In the future, drug targeted approaches based on CD44 expression on CSCs/TICs should be developed, including cytotoxic drugs now employed in clinics (Ghosh et al., 2012). Increasing our understanding of the molecular function of specific CD44 isoforms, the transcriptional regulation of CD44 expression, and the molecular regulation of CD44 alternative splicing will contribute to targeted therapy (Orian-Rousseau and Ponta, 2015; Yan et al., 2015).

### 2.8 Others

Overall, the main players in the field of nanomaterial-assisted lung cancer therapy are summarized. Still some new receptors or therapies are worth giving an update. Hsu et al. (2017) concluded estrogen and its receptor may be a predictor and therapeutic target for lung cancer after evaluating the expression and prognostic effects of estrogen receptor in lung cancer and clinical trials of a combination of estrogen receptor antagonist and EGFR antagonist. Madan et al. (2014) established the feasibility of using estrogen-coupled NPs to target breast cancer cells. Orphan receptors play important roles in development, cellular homeostasis, and diseases, including cancer, and overexpression or underexpression of some receptors has prognostic implications for patient survival, suggesting that they may be novel targets for lung cancer therapy (Safe et al., 2014; Moreno et al., 2018). Currently, immunotherapy has been widely used to treat lung cancer. Lung cancer-borne immunological targets like T-lymphocytes, myeloid-derived suppressor cells (MDSCs), tumor-associated macrophages (TAMs), and dendritic cells (DCs) may be used to modulate tumor activity by targeting various surface-expressed receptors or by interfering with immune cell-specific pathways (Jeanson et al., 2019; Xia et al., 2019; Larionova et al., 2020; Yang et al., 2020; Yu et al., 2020). Nano-drug delivery targeting tumor microenvironment components may enhance the efficacy of immune checkpoint blocking (Kim et al., 2021). Kang et al. (2020) confirmed the anti-tumor effect of T-cell-membrane-coated nanoparticles (TCM NPs) in the treatment of lung cancer in an antigenic non-specific way. The combined application of PD-1/PD-L1 inhibitors and novel platinum-loaded composite NPs has been shown to have a positive synergistic effect (Xue et al., 2021). Cui et al. (2018) demonstrated that glutathione-triggered NPs can enhance the chemotherapy of lung cancer by reconstructing the tumor microenvironment. Zhou et al. (2020) found that the nano-vaccine was very effective in enhancing cell uptake through macrophage phagocytosis, and significantly promoted DCs maturation and antigen presentation.

## 3 Conclusion

To date, we have summarized representative examples of biological targets or receptors regarding lung cancer. Numerous receptors, including VEGFR, integrin, EGFR, FR, TFR, CD44, and σ receptor capable of increasing the specific binding of NPs containing drugs to disease cells, thereby increase the efficacy of chemotherapy. For example, σ ligands induce significant cell death and apoptosis only in tumor tissue, but not in proliferating normal cells such as stem cells (Georgiadis et al., 2017). The weak expression of the TFR in normal lung tissue and the high expression in lung adenocarcinoma tissue indicate that the expression of TFR is related to the histological type of lung cancer, and the expression in lung adenocarcinoma is significantly higher than that in other histological types. Numerous trials have established that TF plays an important role in the abnormal iron metabolism of lung cancer cells; however, the mechanism by which it does so is unknown; we can reduce iron uptake by cancer cells via TF, thereby interfering with the iron metabolism of lung cancer cells, inhibiting lung cancer cell proliferation, and providing new ideas for the treatment of lung cancer diseases. TF has good application prospects in the treatment of tumor diseases. Additionally, the evidence demonstrated that the activity and number of FRs on the membrane surface of a variety of tumor cells (such as ovarian cancer, colorectal cancer, breast cancer, lung cancer, and renal cell carcinoma) were significantly higher than those of normal cells, laying the foundation for the study of drug targeting tumor cells mediated by FA. Reuter et al. (2015) demonstrated that RGD peptide-modified NPs accumulate less in tumors than control PEG-modified NPs. It indicated that researchers should avoid exaggerating the influence of biological ligands. Rather than that, researchers might focus their efforts on receptors to identify more qualified and high-quality ligands. Another important consideration of receptors is the fact that many are overexpressed in tumor cells. We anticipate that specificity comparison trials will become increasingly common as they provide greater benefit in terms of reducing side effects and improving treatment effectiveness. Additionally, several novel targets were investigated that previously received little attention, such as the orphan receptor, bombesin receptor subtype-3 (Moreno et al., 2018).

As we all know, nanotechnology has fundamentally altered the way carriers are used to treat lung cancer and holds enormous possibilities for the future (Parvanian et al., 2017; Sivarajakumar et al., 2018; Mottaghitalab et al., 2019). Physicochemical properties such as size, shape, rigidity, or surface properties, all play a significant role in determining their large-scale distribution (Yoo et al., 2019). NPs of 10–100 nm with a molecular weight above 50 kDa are ideal nanoparticle drug delivery systems (In and Nieva, 2015), considering that tumor blood vessels have wide gap connections with a size of 100–600 nm (Maeda and Matsumura, 1989; Yuan et al., 1995; Maeda, 2012), NPs smaller than 150 nm might escape capture by macrophages in the RES (Aderem and Underhill, 1999; Feelders et al., 1999) and NPs larger than 10nm might avoid leakage into capillaries (Gaucher et al., 2005), but the restriction on size is not absolute and needs to be further studied. It is also important for nanocarriers to overcome the lung’s physiological and anatomical barrier and precisely deliver drugs to tumor cells. Meanwhile, it also faces difficulties in transforming pragmatic medical technology like many new techniques. Additional researches or trials, whether animal or human, are required.

## Glossary

AA: anisamide
AKT: protein kinase B
APT: aptamer
CK: compound K
CSC: cancer stem cell
CSNP: chitosan-based nanoparticle system
DOTAP: 1,2-dioleoyl-3-trimethylammonium-propane chloride salt
DOX: doxorubicin
DTX: docetaxel
EGFR: epidermal growth factor receptor
EPR: enhanced penetration and retention
ER: erlotinib
FR: folate receptor
GSH: glutathione
HA: hyaluronic acid
HIF-1α: hypoxia-inducible factor -1α
LCP: lipid-calcium-phosphate
LP: liposome
LPN: lipid polymer nanoparticle
MMP: matrix metalloproteinase
NLC: nanostructured lipid carriers
NPs: nanoparticles
NRP: neuropilin
NSCLC: non-small cell lung cancer
PBS: phosphate buffered saline
PDI: polydispersity indexes
PGF: placenta growth factor
PLGA: polylactic acid-glycolic acid
PTX: paclitaxel
RES: reticuloendothelial system
RGD: arginine-glycine-aspartic acid
SCLC: small cell lung cancer
SL: soybean lecithin
TFR: transferrin receptor
TIC: tumor initiating cells
VEGF: vascular endothelial-derived growth factor
